# A Text-Book of Histology, Descriptive and Practical

**Published:** 1897-03

**Authors:** 


**A Text-Book of Histology, Descriptive and Practical.** By ARTHUR CLARKSON, M.B. Pp. xx., 554. Bristol: John Wright & Co. 1896.

The aim of the author is to furnish in this book a concise but sufficient description of the minute structure of the organs and tissues of the body, together with an explanation of the methods of preparing the specimens, including the processes of hardening, staining, cutting and mounting, &c. It is illustrated by 174 beautifully drawn and lithographed coloured plates copied from typical sections, most of them remarkably accurate, others intentionally made diagrammatic. But when any departure from the actual specimen is made it is always plainly indicated; and the student can enjoy the great and unusual advantage of learning histology from pictures which are really correct representations of the various structures. Such a combination of theoretical and practical histology cannot, of course, be complete in one moderately-sized volume, but enough is given for all ordinary purposes.

The methods given are the most approved, and the details are described carefully and clearly; great pains have also been taken to enable the reader to understand exactly what he is expected to see in the drawings. It is in these last that the especial interest and excellence of the work lies. There is hardly a plate which is not of great artistic and scientific merit. We only regret that there are not a few more drawings to make the book more complete. We would suggest, for example, that some representations of specimens of nerve-tissue stained by Golgi's method, some plates of the various nerve-endings, and some of the modern methods of staining the white corpuscles of the blood, &c., might be included in another edition.

Here and there we think the descriptive portions might be made a little clearer for the beginner; but whatever fault is to be found with this is more than counterbalanced by the clearly-drawn and well-coloured plates, which speak very plainly for themselves. The semi-diagrammatic representations of liver, lung, lymph-channels, kidney, &c., are very helpful, and add to the value of the book for teaching purposes.

